# Mental toughness and self-efficacy of elite ultra-marathon runners

**DOI:** 10.1371/journal.pone.0241284

**Published:** 2020-11-04

**Authors:** Anthony W. Brace, Kendall George, Geoff P. Lovell

**Affiliations:** 1 School of Health and Behavioural Sciences, University of the Sunshine Coast, Sippy Downs, Australia; 2 School of Nursing, Midwifery and Paramedicine, University of the Sunshine Coast, Sippy Downs, Australia; 3 Department of Sport, Hartpury University, Gloucester, United Kingdom; University of Pittsburgh, UNITED STATES

## Abstract

Minimal research has examined psychological processes underpinning ultra-marathon runners’ performance. This study examined the relationships between mental toughness and self-efficacy with performance in an elite sample of ultra-marathon runners competing in the 2019 Hawaiian Ultra Running Team’s Trail 100-mile endurance run (HURT100). The Mental Toughness Questionnaire (SMTQ) and the Endurance Sport Self-Efficacy Scale (ESSES) were completed by 56 elite ultra-marathon runners in the HURT100 (38 males, 18 females; *M*_age_ = 38.86 years, *SD*_age_ = 9.23). Findings revealed mental toughness and self-efficacy are highly related constructs (*r*(54) = 0.72, *p* < 0.001). Mental toughness and self-efficacy did not significantly relate to ultra-marathon performance (mental toughness and self-efficacy with Ultra-Trail World Tour (UTWT) rank *F*(2, 53) = 0.738, *p* = 0.483; mental toughness and self-efficacy with likelihood would finish the HURT100 χ^2^ = 0.56, *p* = 0.756; mental toughness and self-efficacy with HURT100 placing and time *F*(2, 53) = 1.738, *p* = 0.186 and *F*(2, 30) = 2.046, *p* = 0.147, respectively). However, participants had significantly and meaningfully higher mental toughness (*M* = 45.42, *SD* = 4.26, medium and large effect sizes) than athletes from other sports previously published. Our interpretation is that these results taken in conjunction, suggest a threshold of mental toughness that performers require to be of the standard needed to be able to prepare for and compete in elite ultra-marathon events such as the HURT100; once this mental toughness threshold is met, other factors are likely to be more influential in determining elite level ultra-marathon performance.

## Introduction

*Ultra-endurance* events have been described as prolonged periods of physical activity covering further distance than the standard marathon (42.195 km) or lasting more than 6 hours, typically involving running, cycling, or swimming [1, 2]. Ultra-endurance events that are solely completed on foot are generally termed *ultra-marathon* events. Ultra-marathon events often range from 50 to 100 km, however, ultra-marathon races do span much further than this and commonly involve challenging and tumultuous terrain, e.g., the Hawaiian Ultra Running Trail 100-Mile Endurance Run (HURT100) (161 km), Bad Water 135 (217 km), and the 6693 Ultra (566 km). Ultra-endurance and ultra-marathon competition is gaining popularity, with the number of participants growing over the last decade, especially so for women [3]. Although globally the ultra-endurance and ultra-marathon participation rates are growing, research that specifically focusses on the people that engage in this unique and extreme form of physical activity is limited. Of the research that has considered ultra-endurance and ultra-marathon, the majority has focused on demographic characteristics [3, 4], physiological demands (e.g., gastrointestinal problems, lower limb injuries, and debilitating muscle cramping, [5]), and changes in diet and physical activity across participants’ lifetimes [6, 7]. However, there is minimal research that specifically focuses upon the psychological aspects of ultra-endurance and ultra-marathon, examining how these challenging feats of endurance are achieved, with even less research investigating elite level performers.

To gain further understanding as to how athletes overcome the challenges associated with ultra-endurance and ultra-marathon competition, further psychology focused research is warranted. Likely beneficial and insightful perspectives to consider these psychological challenges is the concept of mental toughness. Mental toughness is a psychological concept that has been shown to be an important construct in sport [8]. Gucciardi et al. (2015) [8] define mental toughness as “a personal capacity to produce consistently high levels of subjective (e.g., personal goals or strivings) or objective performance (e.g., sales, race time, GPA) despite everyday challenges and stressors as well as significant adversities” (p.28). While mental toughness has only received very limited consideration in quantitative studies of ultra-endurance and ultra-marathon, previous research has concluded mental toughness to be an important factor for success in mixed martial arts [9], football (soccer) [10], tennis [11], hockey [12], Australian football [13], cricket [14], rugby league [15], and endurance athletes [16]. Further supporting the expected saliency of mental toughness for ultra-endurance and ultra-marathon competition is evidence that mental toughness components are reported to be higher in individuals who are able to endure physical exertion for longer periods [17].

Numerous conceptual models have been posited to explain mental toughness (e.g., [13, 18, 19]), however it appears no clear consensus has been reached regarding this conceptualization [20]. For this investigation, we have adopted the Goal-Expectancy-Self-Control model [20] as a guiding theoretical framework. The Goal-Expectancy-Self-Control model, developed from a critical review of mental toughness research, suggests that following presentation of a stressor three psychological resources characterise mental toughness: self-control, goals, and self-efficacy. Self-efficacy was thus selected as a variable of focus for this investigation based on its inclusion in the Goal-Expectancy Control model, and that additionally self-efficacy has consistently been shown to be associated with sporting performance and success [21–24]. Bandura (1982) [25] suggests “perceived self-efficacy is concerned with judgements of how well one can execute courses of action required to deal with prospective situations” (p.122). Furthermore, limited self-efficacy is associated with athletes applying less effort when they perceive they cannot successfully complete a task, as opposed to focusing their efforts upon mastering the task [25]. Thus, ultra-endurance and ultra-marathon athletes with limited self-efficacy can be considered to be less likely to persist, perform, and complete, compared to those with higher levels of self-efficacy. If it is assumed that prolonged physical activity is likely to be associated with increased pain and discomfort, self-efficacy may also offer additional benefits through analgesic effects. Self-efficacy has been reported to be associated with endogenous opioids, buffering pain while enduring high levels of discomfort [5, 26, 27].

With regard to mental toughness, only minimal research has investigated ultra-marathon athletes, much of which has been qualitative, and predominately considered in samples other than runners [26, 28]. Zeiger and Zeiger (2018) [16], who did use a quantitative methodology and did include some ultra-marathon participants, endeavoured to analyse the underlying structures of mental toughness using latent profile analysis of the *Sports Mental Toughness Questionnaire* (SMTQ [29]). The key findings from Zeiger and Zeiger (2018) [16] were associations between mental toughness and satisfaction with race results, gender, and age. The mental toughness subscale self-belief, as measured by the PPI-A [30], had the largest effect size between different mental toughness classes (High mental toughness, Moderate mental toughness, Low mental toughness), with greater self-belief trending towards greater mental toughness. Also, high-division placement positively associated with higher mental toughness [16]. Zeiger and Zeiger’s (2018) [16] findings do progress our understanding of mental toughness in endurance athletes, however, limitations of their study include lack of focus on ultra-marathon participants, recollected data, and a sample that unlikely generalises to elite populations.

While there appears to be reliable evidence from a range of sportsthat mental toughness and self-efficacy do associate with sporting success, and logical proposition of why mental toughness and self-efficacy should contribute to ultra-marathon performances, there is minimal research concerning these psychological constructs in ultra-marathon runners. This dearth in our understanding of how mental toughness and self-efficacy quantitatively pertain to ultra-marathon limits the evidence-based design of efficacious applied interventions to enhance ultra-marathon performance through the manipulation of mental toughness and self-efficacy. In response to the salience of this topic area, increased ultra-marathon participation rates, yet limitations in current understanding, the overarching aim of this research was to quantitatively assess how mental toughness and self-efficacy relate to elite ultra-marathon performance. To approach this aim, five specified objectives were examined in a sample of elite ultra-marathon participants with data gathered temporally proximal to competition: (1) How mental toughness and self-efficacy relate to each other as constructs, as well as to the SMTQ subscales of Confidence, Constancy, and Control; (2) Whether mental toughness and self-efficacy contribute to Ultra-Trail (ultra-marathon) World Tour rankings [31]; (3) How mental toughness and self-efficacy predict ultra-marathon completion (specifically the HURT100); (4) Whether mental toughness and self-efficacy predict absolute (i.e. finishing time and placing) ultra-marathon performance (specifically in the HURT100); and (5) If mental toughness and self-efficacy in elite ultra-marathon athletes is higher than athletes from other sports. With regard to expected results, it was hypothesized that overall mental toughness and the subscales of the SMTQ (Confidence, Constancy, and Control) would significantly relate to self-efficacy. That mental toughness and self-efficacy would positively correlate Ultra-Trail World Tour ranking, and mental toughness and self-efficacy would predict whether an ultra-marathon athlete would finish the HURT100. Furthermore, that performance measures of HURT100 placing and time would significantly associate with mental toughness and self-efficacy. Finally, mental toughness would be significantly greater in ultra-marathon athletes compared to athletes from other sports.

## Method

The design of this investigation was a quantitative, non-experimental, cross-sectional, analysis of self-reported variables.

### Participants and recruitment

Following institution ethical approval (University of the Sunshine Coast Human Research Ethics Committee; approval number A181188) and informed consent, 140 elite level ultra-marathon athletes over 18 years of age competing as individuals in the HURT100 were invited on a voluntary basis to complete an online survey hosted on SurveyMonkey. The HURT100 is a 100 mile (161 km) running competition through the Hawaiian mountains. The HURT100 course has an elevation change of 7,620 meters gain and loss over for the entirety of the 100 mile course. The race is spread over five 32.19 km laps, with each lap containing three aid stations for athlete re-hydration, nutrition, and medical attention. The HURT100 is a highly regarded international event to the ultra-marathon community; in 2019 all but two competitors were listed on the Ultra-Trail World Tour rankings, justifying the contention that HURT100 athletes are elite [32].

Recruitment was conducted through email of all individual HURT100 ultra-marathon competitors. Inclusion criteria required athletes to be over the age of 18, competing in the 2019 HURT100, with, fluent reading and comprehension of English. Of the 140 competitors invited to complete the survey, 41% provided meaningful data (*N* = 56, *M*_age_ = 38.86, *SD*_age_ = 9.23, %_male_ = 67.9).

### Materials and procedure

Participants completed the online survey as part of a larger program of investigation. Other collected data represented athletes’ qualitative experiences of participating in the HURT 100 related to mental toughness, pain tolerance, and factors contributing to race outcome (we intend to publish these qualitative data in a separate publication). The survey included measures of mental toughness, self-efficacy, and demographic data (age, gender, training experience, world ranking, training hours per week, occupation, employment, and education). The survey was made available to participating athletes 12 days prior to the start of the HURT100, closing immediately prior to the start; the mean average duration between completion of the survey race start was 6.16 days (*SD* = 2.54).

#### Sports Mental Toughness Questionnaire (SMTQ)

Mental toughness was assessed by the 14 item Sports Mental Toughness Questionnaire (SMTQ [29]), providing an overall sports mental toughness score and three subscales; Confidence, Constancy, and Control [29]. SMTQ overall mental toughness scores have a possible range from 14–56, with higher scores representing higher levels of mental toughness. Confidence scores range from 6–24, with Constancy and Control scores ranging from 4–16. Previous research has reported that the SMTQ is a valid and reliable measure as evidenced by acceptable internal consistency scores (Cronbach alpha; Confidence 6 items; *a* = 0.80, Constancy 4 items; *a* = 0.74, and Control 4 items; *a* = 0.7 [29]). For the current sample, internal consistency (Cronbach alpha) for the entire questionnaire was also considered acceptable (14 items; *a =* 0.65), as too was for the subscale of Control (4 items; *a =* 0.65). Cronbach alpha scores for the subscales of Confidence (6 items; *a =* 0.52) and Constancy (4 items; *a =* 0.42) were low. However, acknowledging the acceptable internal consistency score for the whole scale, the reported negative effect of limited item numbers upon Cronbach alpha, and the desire to maintain the structural integrity of the previously validated and commonly adopted scale, the decision was made to accept the internal consistency scores both the Confidence and the Constancy sub-scales [33–36].

#### Endurance Sports Self-Efficacy Scale (ESSES)

Self-efficacy was measured by the Endurance Sport Self-Efficacy Scale (ESSES; [37]) The ESSES is a 11-item measure of sport specific self-efficacy with an emphasis on endurance sports. Possible ESSES scores range from 0–100 with higher scores representing greater levels of self-efficacy. The ESSES has been reported to a valid and reliable measure of self-efficacy (11 items; *a* = 0.88 [37]). ESSES internal consistency scores for the current sample were acceptable (11 items; *a =* 0.85 [38]).

#### HURT100 2019 performance

Performance was assessed as finishing place and finishing time for the HURT100 2019; these data were obtained from the official event website. The measure of HURT100 2019 completion was based on whether participants completed the event in accordance with the event rules and regulations.

### Data analysis

All statistical analyses were performed using SPSS (version 24, SPSS Inc., Chicago, IL). Prior to statistical analysis data were checked for normality and that relevant assumptions were met. Normality was assessed through visual inspection of histograms and normal P-P plots [38]. Linearity was assessed through visual inspection of *ZRESID against *ZPRED plots and homoscedasticity was found to be acceptable [39]. While multicollinearity is always a concern when variables are correlated, as all variance inflation factor (VIF) scores were well below 10 with tolerance statistics all above 0.2, can confidently conclude that there was no collinearity within the data. Eigen values were assessed to further indicate the assumption of collinearity was acceptable [38]. Durbin Watson tests indicated the independence of errors assumption was met [38]. Specific statistical analyses for each research objective are presented in the results section. For all analysis, significance was set at the 95% level of confidence. G-Power sample size estimates for the regression analyses to obtain 0.80 power, with Cohen’s *d* (39) effect size estimates of 0.2 (small effect size), with two predictors, indicated that a sample of at least 52 was required. Effect sizes for Pearson’s bivariate correlation (research objective one) were based on calculated *r* values, with *r* = 0.10 considered small effect, *r* = 0.30 considered medium effect, and *r* = 0.50 considered large (38). Effect sizes for regression analysis (research objectives one, two, three, and four) were based on Cohen’s ƒ^2^, with ƒ^2^ = 0.10 considered small effect, ƒ^2^ = 0.25 considered medium effect, and ƒ^2^ = 0.35 considered large (39). Effect sizes for single sample *t*-tests (research objective five) were based on Cohen’s *d*, with *d* = 0.20 considered small effect, *d* = 0.05 considered medium effect, and *d* = 0.80 considered large effect (39).

## Results

The sample consisted of 56 participants: 68% male; 37% between 24–34 years of age, 37% between 35–44, and 25% between 45–65 (*M*_age_ = 38.86, *SD*_age_ = 9.23). Fifty-five percent of participants had less than 5 years experience of running ultra-marathon, 30% had between 5–10 years of experience, and 14% had over 10 years’ experience of running ultra-marathon. Participant characteristics can be found in Table 1, descriptive statistics and intercorrelations of measured variables are presented in Table 2.

**Table 1:** *Characteristics of* Ultra-Marathon *runners*.

Variable	Characteristic	*n*	%
Gender	Male	38	67.9
	Female	18	32.1
Age	24–34	21	37.5
	35–44	21	37.5
	45–65	14	25
Education	< Secondary School	2	3.6
	Secondary School	8	14.3
	College Certificate/Diploma	4	7.1
	Bachelor’s Degree	19	33.9
	Advanced Degree	23	41.1
Years Competing in ultra- marathon	< 5 years	21	37.5
	5–10 years	27	48.2
	> 10 years	8	23.2
Hours Training Per Week	6–10 Hours	16	28.6
	11–15 Hours	27	48.2
	16+ Hours	13	23.2
Ultra-Trail World Tour Rank	40–59	5	9.26
	60–79	24	44.44
	80–100	25	46.3
Placing in the 2019 HURT100	Top 10	6	10.70
	11–20	6	10.70
	21+	21	37.50
	Did not finish	23	41.10

**Table 2:** Descriptive statistics and intercorrelations between measured variables (n = 56).

Variable	1	2	3	4	5	6	7	8	*M (SD)*
1. SMTQ: Total Average	*--*								45.42 (4.26)
2. SMTQ: Confidence	0.802**	*--*							18.50 (3.11)
3. SMTQ: Constancy	0.509**	0.223	*--*						15.00 (1.20)
4. SMTQ: Control	0.546**	0.019	0.128	*--*					11.92 (2.11)
5. ESSES: Total Average	0.720**	0.584**	0.215	0.470**	*--*				83.08 (9.48)
6. Ultra-Trail World Tour 2018 Rank^†^	0.184	0.065	0.216	0.184	0.232	*--*			73.78 (18.78)
7. HURT100 2019 time (hrs)	-0.327	-0.261	-0.233	-0.020	-0.301	-0.749**	*--*		30.90 (3.68)
8. HURT100 2019 position^†^	-0.252	-0.184	-0.131	-0.074	-0.220	0.123	0.808**	*--*	18.48 (21.37)

*Note*. World ranking and HURT100 2019 finishing positions correlations based on separate sex classification results

*p < 0.5

**p < 0.01

^†^Spearman’s rho correlations.

To analyse research objective one, Pearson’s bivariate correlation assessed association between mental toughness and self-efficacy, additionally, one linear multiple regression was employed to determine associations between self-efficacy and mental toughness SMTQ subscales (confidence, control, and constancy). Results to research objective one demonstrated that mental toughness as measured by the SMTQ and self-efficacy as measured by the ESSES were strongly correlated (*r*(54) = 0.72, *p* < 0.001) with large effect size. Multiple linear regression demonstrated a significant relationship between the SMTQ subscales confidence, control, and constancy with self-efficacy, explaining 52.7% of self-efficacy variance (*F*(3, 52) = 21.41, *p* < 0.001, *R*^2^ = 0.55, adjusted *R*^2^ = 0.53, with large effect size, ƒ^2^ = 1.24 [38]). SMTQ-confidence made the largest significant unique contribution to self-efficacy (β *=* 0.57, *t* = 5.97, *p <* 0.001, 95% CI [1.15, 2.32]), explaining 31% of self-efficacy variance, followed by SMTQ-control (β *=* 0.46, *t* = 4.87, *p <* 0.001, 95% CI [1.20, 2.89]), explaining 20% of self-efficacy variance. SMTQ-constancy failed to make a significant contribution to self-efficacy. Descriptive statistics for the SMTQ and ESSES can be found in Table 2.

To explore research objective two, multiple linear regression was used to assess how mental toughness and self-efficacy related to Ultra-Trail World Tour rank. Results revealed than neither mental toughness nor self-efficacy, *F*(2, 53) = 0.738, *p* = 0.483, significantly related to Ultra-Trail World Tour rank. To analyse research objective three, a binary logistic regression was undertaken to explore the association between mental toughness and self-efficacy with the likelihood of completing the HURT100 2019. Results indicated mental toughness and self-efficacy did not significantly relate the likelihood an athlete would finish the HURT100 (χ^2^ = 0.56, *p* = 0.756). In response to research objective four, two separate linear and multiple regressions were performed to determine the relationship between self-efficacy and mental toughness with HURT100 2019 performance (placing and time). Results indicated mental toughness and self-efficacy did not significantly relate to placing nor time measures of performance (*F*(2, 53) = 1.738, *p* = 0.186 and *F*(2, 30) = 2.046, *p* = 0.147 respectively).

For research objective five, single sample *t*-tests compared mental toughness in the current sample of elite ultra-marathon runners with previously published mental toughness SMTQ data for athletes from other sports. Results (Table 2) demonstrated the current sample of ultra-marathon runners () had significantly and meaningfully greater mental toughness (SMTQ; *M* = 45.42, *SD* = 4.26) than; high level adolescent female hockey players (*M* = 41.9, *SD* = 5.4, *t*(55) = 6.19, *p* < 0.001, *d* = 0.83, 95% CI [2.39, 4.67]) [12], professional Welsh football (soccer) players (*M* = 42.00, *SD* = 8.37, *t*(55) = 6.01, *p* < 0.001, *d* = 0.80, 95% CI [2.28, 4.57]) [10], professional mixed martial artists (*M* = 42.80, *SD* = 5.64, *t*(55) = 4.61, *p* < 0.001, *d* = 0.61, 95% CI [1.48, 3.77]) [9], South African tennis players (*M* = 41.22, *SD* = 4.67, *t*(55) = 7.39, *p* < 0.001, *d* = 0.98, 95% CI [3.06, 5.35]) [11], high-performing adolescent male athletes (*M* = 42.16, *SD* = 4.87, *t*(55) = 5.73, *p* < 0.001, *d* = 0.76, 95% CI [2.12, 4.41]) [40] (values are presented to the number of decimal places provided in the previously published data; for Wieser et al, [10], as the group average SMTQ was presented as a percentage, we multiplied the reported percentage by the maximum possible score to provide an absolute SMTQ score). Descriptive statistics for t-test comparisons are presented in Table 3.

**Table 3:** Descriptive and inferential statistics for single sample t-tests between current Ultra-Marathon runners sample and previously published SMTQ data for different sports.

Sport	Descriptive statistics			Inferential statistics			
	*n*	*M*	*SD*	*t(df)*	*p*	95% CI	Cohen’s *d*
Ultra-Marathon Elite Athletes (current sample)	56	45.42	4.26	*--*	*--*	*--*	
Adolescent Female Hockey Players [12]	484	41.9	5.4	6.19(55)	< 0.001*	[2.39, 4.67]	0.83
Professional Welsh Football [10]	20	42.00	8.37	6.01(55)	< 0.001*	[2.28, 4.57]	0.80
Professional Mixed Martial Artists [9]	49	42.80	5.64	4.61(55)	< 0.001*	[1.48, 3.77]	0.61
South African tennis [11]	365	41.22	4.67	7.39(55)	< 0.001*	[3.06, 5.35]	0.98
High-performing adolescent male athletes [40]	151	42.16	4.87	5.73(55)	< 0.001*	[2.12, 4.41]	0.76

*Note*.

*p < 0.001.

## Discussion

Minimal quantitative research has specifically focused on ultra-marathon runners and the associated psychological factors required for success in ultra-marathon. Therefore, the purpose of this study was to analyse the psychological constructs of mental toughness and self-efficacy within ultra-marathon runners to determine how these concepts relate to each other and to ultra-marathon performance.

In answer to research objective one, it was found that mental toughness and self-efficacy do correlate strongly with each other (*r*(54) = 0.72, *p* < 0.001); as was expected due to concept relatedness in the literature [8, 20, 41, 42]. This finding further confirms that mental toughness is multidimensional and shares conceptual space with other constructs. Furthermore, the subscale of the SMTQ Confidence was found to explain 31% of the variation within self-efficacy. As self-efficacy has been defined as a belief in one’s own abilities [25], it can be suggested that having confidence as opposed to self-doubt [30] would contribute to self-efficacy.

One possible explanation as to why mental toughness relates to self-efficacy is due to perceived self-efficacy being linked to endogenous opioid activation [27]. High levels of self-efficacy may produce greater levels of mental toughness due to endogenous opioids buffering pain; as mental toughness has been linked to pain tolerance [43], self-efficacy opioid activation may affect mental toughness. Athletes could be experiencing higher levels of mental toughness because they have a greater ability to buffer pain through the body's endocrine system. Studying the relationship between the body’s pain buffering systems and physiological toughness (i.e. an increase in active opioid receptors) could be beneficial to understanding the connection between mental toughness and self-efficacy.

With regard to research objectives two, three, and four, Ultra-Trail World Tour rank, finishing the HURT100, and performance were not significantly associated with levels of mental toughness and self-efficacy. This finding was contrary to our predictions. However, of the limited previous research that has demonstrated positive significant relationship between self-efficacy and completion of ultra-marathon type events [42], despite relatively large sample size only small effect sizes were observed. Our proposed explanation of these non-significant observed relationships of self-efficacy and mental toughness with performance, and the only small effects previous reported [42], relates to our finding for research objective five. The results of one-sample *t*-tests for objective five demonstrated that mental toughness in the current sample was significant and meaningfully higher than mental toughness previously reported for other sports performers. Our interpretation is that these results taken in conjunction, suggest a threshold of mental toughness that performers require to be of the standard needed to be eligible to prepare for and complete in elite ultra-marathon events such as the HURT100; once this mental toughness threshold is met, other factors are likely to be more influential in determining performance. This does not negate the importance of mental toughness for ultra-marathon performance, but does highlight a non-linear relationship between mental toughness and performance, as well as emphasizing the likely multifaceted nature of factors that determine ultra-marathon performance. However, further research should consider this conclusion, contrasting elite ultra-marathon athlete mental toughness and self-efficacy scores with those of ‘sub-elite’ ultra-marathon runners or those participating in shorter distances.

In application of these conclusions that higher categories of ultra-marathon competition require supra-threshold levels of mental toughness and associated self-efficacy, psychological skills training focused on increasing mental toughness and self-efficacy could potentially help in improving the likelihood an athlete would advance into the highest category of ultra-marathon performance. While psychological skills training has been shown to improve athletes’ performance in other sports [43, 44], there is very limited research that has considered the effects of mental toughness and self-efficacy training programs in ultra-marathon samples. Future research should also consider the development of mental toughness and self-efficacy training programs and interventions specific to the somewhat unique challenges of ultra-marathon. Qualitative research that considers the reported challenges, barriers, and enablers to ultra-marathon performance would be key in informing the design of such ultra-marathon specific mental toughness and self-efficacy programs and interventions.

This study has limitations. The use of a mental toughness assessment tool that is not specific to ultra-marathon is likely to limit these results. The SMTQ was constructed from a sample of athletes other than ultra-marathon runners, thus introducing the potential that key aspects of mental toughness in ultra-marathon could be undetected. Development of a mental toughness tool specific to ultra-marathon would appear to have utility in both research and applied practice. A similar limitation related to self-efficacy is also likely to exist. Additionally, this research was based on participant self-report, a methodology that does contain challenges related to participants having sufficient knowledge of their own thoughts to be able to accurately report, as well response distortion due social desirability bias [45]. Furthermore, while the size of this purposefully elite sample was in excess of the power analysis predicted required sample size (to obtain 0.8 power assuming a small 0.2 effect size) there does remain the risk of a type II error.

In conclusion, this investigation provided novel findings demonstrating that ultra-marathon runners measure significantly higher than other sports in mental toughness. While mental toughness and self-efficacy strongly relate to each, mental toughness and self-efficacy do not relate to performance above a super-threshold; although a criterion level appears requisite to be able complete the elite level, potentially associated with the ability to manage and complete the required extensive training load. Once this criterion level of mental toughness and self-efficacy is satisfied, other factors, potentially including other psychological factors as well as physical and logistic, appear to have greater saliency in determining elite ultra-marathon performance outcomes.
